# First Record of *Fusarium verticillioides* as an Entomopathogenic Fungus of Grasshoppers

**DOI:** 10.1673/031.011.7001

**Published:** 2011-05-29

**Authors:** SA Pelizza, SA Stenglein, MN Cabello, MI Dinolfo, CE Lange

**Affiliations:** ^1^ Instituto de Botánica Carlos Spegazzini, Facultad de Ciencias Naturales y Museo, Universidad Nacional de La Plata; ^2^ Centro de Estudios Parasitológicos y de Vectores (CEPAVE), CCT-La Plata-CONICET-UNLP, Calle 2 # 584, La Plata (1900), Argentina; ^3^ Laboratorio de Biología Funcional y Biotecnología (BIOLAB)-CEBB-CONICET, Facultad de Agronomía de Azul, UNCPBA, Republica de Italia # 780, Azul (7300), Argentina

**Keywords:** Acrididae, Orthoptera, *Ronderosia bergi*, *Tropidacris collaris*, Chaco Province, Argentina

## Abstract

*Fusarium verticillioides* (Saccardo) Nirenberg (Ascomycota: Hypocreales) is the most common fungus reported on infected corn kernels and vegetative tissues, but has not yet been documented as being entomopathogenic for grasshoppers. Grasshoppers and locusts represent a large group of insects that cause economic damage to forage and crops. *Tropidacris collaris* (Stoll) (Orthoptera: Acridoidea: Romaleidae) is a large and voracious grasshopper that in recent years has become an increasingly recurrent and widespread pest in progressively more greatly extended areas of some of in Argentina's northern provinces, with chemical insecticides being currently the only means of control. During February and March of 2008–09, nymphs and adults of *T. collaris* were collected with sweep nets in dense woodland vegetation at a site near Tres Estacas in western Chaco Province, Argentina, and kept in screened cages. *F. verticillioides* was isolated from insects that died within 10 days and was cultured in PGA medium. Pathogenicity tests were conducted and positive results recorded. Using traditional and molecular-biological methods, an isolate of *F. verticillioides* was obtained from *T. collaris*, and its pathogenecity in the laboratory was shown against another harmful grasshopper, *Ronderosia bergi* (Stål) (Acridoidea: Acrididae: Melanoplinae). The mortality caused by *F. verticillioides* on *R. bergi* reached 58 ± 6.53% by 10 days after inoculation. This is the first record of natural infection caused by *F. verticillioides* in grasshoppers.

## Introduction

The genus *Fusarium* comprises a large group of species of filamentous fungi widely distributed in soil usually in association with plants. Most species are saprotrophic and relatively abundant members of the soil microbiota (Leslie and Summerell 2006). Many *Fusarium* species are well-known as pathogens of plants, insects, and humans (Majumbar et al. 2008), although there are *Fusarium* species that are insect pathogens but are not pathogenic to the plant (Kuruvilla and Jacob 1979a, 1979b, 1980). More than 13 *Fusarium* species are pathogenic to insects, and the genus has a host range that includes Coleoptera, Diptera, Hemiptera, Hymenoptera and Lepidoptera (Teetor-Barsch and Roberts 1983; Humber 1992). *Fusarium* includes various species/strains that are able to produce potent secondary metabolites, such as trichothecenes (Kilpatrick 1961; Kuno and Ferrer 1973), fumonisins (Kuruvilla and Jacob 1979a) and beauvericin (Kuruvilla and Jacob 1979b, 1980; Gupta et al. 1991). The latter is a widespread metabolite among entomopathogenic fungi, such as *Beauveria bassiana* and *Paecilomyces fumosoroseus* (Leath and Newton 1969; Loke et al. 1970). *Fusarium verticillioides* (Saccardo) Nirenberg (Ascomycota: Hypocreales) is often the most common fungus reported from infected corn kernels and vegetative tissues (Foley 1962; Kommedahl and Windels 1981; Nelson et al. 1993; Kedera et al. 1999; Desjardins et al. 2000). In Argentina, its presence has been registered in corn (Peiretti-Uzal et al. 2007) but has not been recorded as an entomopathogen. In France *F. verticillioides* (as *Fusarium moniliforme*) was isolated from *Ostrinia nubilalis* (Vago 1958) and *Bombyx mori* (Vago and Nicot 1954). *Fusarium acridiorum* (Thabut) (=*Trichothecium acridiorum*) is a cuticular parasite of the desert locust *Schistocerca gregaria* (Akbar et al. 1958), which constitutes the only previous record of a species of *Fusarium* isolated from the Acridoidea.

As in other regions of the world, grasshoppers and locusts are important agricultural pests in different parts of Argentina. Damage to forage and a variety of crops have been reported from at least 15 of the 201 species known for the country (Lange et al. 2005; Carbonell et al. 2006). Eight of the harmful species are melanoplines (Acrididae, Melanoplinae) and three romaleids (Romaleidae: Romaleinae). The romaleid *Tropidacris collaris* (Stoll) (Orthoptera: Acridoidea: Romaleidae) has become in recent years an increasingly recurrent and extended pest in some of the northern provinces, particularly in parts of Córdoba, Santiago del Estero, and Chaco. *Tropidacris collaris*, one of the largest grasshoppers known (♂ = 73–101 mm, ♀ = 92–126 mm), is strongly gregarious during juvenile development and it is voracious. Although adults tend to prefer hard-leaf trees and bushes, *T. collaris* is actually a polyphagous species (Barrera and Paganini 1975; Carbonell 1986). The bands of nymphs consume virtually all available plant material. Currently, chemical insecticides are the only mean of control. Pathogens have not been reported, and *Paranosema locustae*, a microsporidium developed in the USA as a biocontrol agent of grasshoppers that was introduced and became established in some other areas of the country (Lange and Azzaro 2008), did not produce encouraging results when tested against *T. collaris* under laboratory conditions (Lange et al. 2008).

Here we report obtaining, by traditional and molecular methods, an isolate of *F. verticillioides* in *T. collaris*, and its pathogenecity in the laboratory (under controlled conditions) against another harmful grasshopper, the melanopline *Ronderosia bergi* (Stål).

## Materials and Methods

During February and March of 2008–09, nymphs and adults of *T. collaris* were collected with sweep nets in dense woodland vegetation at a site (27° 8′ 21.9″ S; 61° 34′ 23.8″ W) near Tres Estacas in western Chaco Province, Argentina. The area is within the Chaqueña biogeographic province (Cabrera and Willink 1973), where the annual rainfall is 600 mm and the average temperatures range between 19 and 21 °C with summer maxima reaching 42–46 °C. The samples were immediately taken to the laboratory where the grasshoppers were kept in groups in wirescreened cages in a rearing room under controlled conditions (30 °C, 14:10 light-dark photoperiod, 60% relative humidity). This setting usually favors the expression of entomopathogenic fungi present in field-collected, infected insects (Shah et al. 1997).

**Figure 1.**
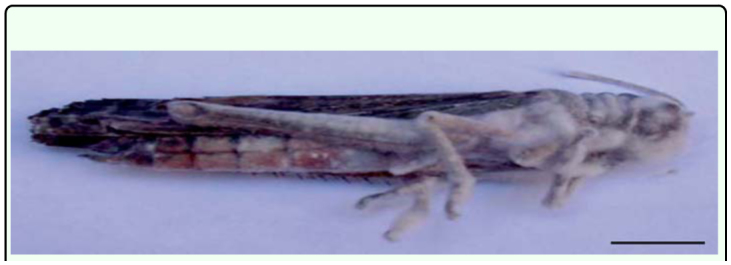
Adult *Tropidacris collaris* 48 h after its death caused by *Fusarium verticilliodes.* Scale bar: 14 mm. High quality figures are available online

The grasshoppers that died within 10 days after collection were superficially sterilized by placing the specimens in 70% ethanol for a few seconds, then washed in sterile distilled water, followed by 0.5% sodium hypochlorite for 1 min, and rinsed again in sterile distilled water according to Lacey and Brooks (1997). They were then placed in a sterile culture chamber consisting of a Petri dish (150 mm diameter) with a filter-paper disk that was periodically moistened with sterile distilled water and incubated at 25° C in the dark. Daily checks were performed during the first 5 days post-mortem. The filamentous fungi emerging from the dead individuals (Figure 1) were transferred to Petri dishes containing potato-dextrose agar (PDA) + antibiotics, and incubated (26° C). The fungal species isolated from *T. collaris* were identified on the basis of the macromorphological appearance of the colonies—such as color, diameter, mycelial texture—and their micromorphological characteristics were observed under phase-contrast microscopy. Additionally, a specific PCR was performed to confirm the fungal species. For DNA extraction the isolate was grown on PDA medium at 25 ± 2 °C under a 12 h light-dark photoperiod and 6 day-old cultures were quantitatively scraped from the surfaces of three Petri dishes with a scalpel, frozen in liquid nitrogen, and then ground into a fine powder in a mortar. Genomic DNA was extracted by the so-called CTAB method described by Stenglein and Balatti (2006). A *F. verticillioides*-specific PCR was performed with primers 5′-GTCAGAATCCATGCCA GAACG-3′-forward and 5′- CACCCGCAGC AATCCATCAG-3′- reverse (Patiño et al. 2004). The amplification was carried out in a 25-µl final volume containing 12–15 ng of genomic DNA, 10X reaction buffer (2 mM Tris-HC1 pH 8.0, 10 mM KC1, 0.01 mM EDTA, 1mM DTT, 50% [v/v] glycerol, 0.5% [v/v] Tween 20, 0.5% [v/v] Nonidet P40), 0.5 µM of each primer, 200 µM of each dNTP (Genbiotech S.R.L.), 2.5 mM MgC12, and 1.25 units of Taq DNA polymerase (Genbiotech, www.genbiotech.com). DNA amplification was performed in an XP thermal cycler (Bioer Technology Co., www.bioer.com.cn) with an initial denaturing step at 95° C for 2 min; followed by 29 cycles at 95° C for 30 s, 54° C for 35 s, and 72° C for 45 s; and a final extension cycle at 72° C for 2 min. The specific product of 800 bp was examined by electrophoresis in 1.5% (w/v) agarose gels containing GelRedTM (Genbiotech) at 80 V in 5X Trisborate-EDTA buffer for 3–4 h at room temperature. The fragment was visualized under ultraviolet light. The size of the DNA fragment was estimated by comparing the DNA band with a 100-bp DNA ladder (Genbiotech).

The fungal isolate was deposited at the Fungal Culture Collection of the Spegazzini Institute of Botany as LPS 1057. The viability of the conidial fungi was determined after 24 hours by means of the techniques described by Lane et al. (1988). The germination test was repeated for each stock suspension to increase the accuracy of the viability assessments.

Our efforts to develop a breeding colony of *T. collaris* were unfortunately not successful (Lange et al. 2008). Thus, the insects used for testing pathogenicity in this study were *R. bergi*, bred in a grasshopper colony at CEPAVE.

Three replicates (on different dates) of 50 third-instar nymphs each of healthy *R. bergi* were sprayed in groups of 10 with 1,000 µl of a suspension of 2.8 × 10^6^ conidia/ml (in 0.01% [v/v] Tween 20) according to Majumbar et al. (2008). The conidia had been harvested after cultivation on PGA for 10 days at 25 °C in the dark. Three additional replicates of 20 grasshoppers each (in two groups of 10) were sprayed with 1,000 µl of 0.01% [v/v] Tween 20 for use as controls. The grasshoppers were maintained in groups of 10 in acetate tubes (50 × 9 cm) after (Henry 1985) and fed with lettuce (*Lactuca sativa* L.) leaves. Treated and control insects were maintained at 30 °C, 60% relative humidity, and a 14:10-h light-dark photoperiod. Cumulative mortality was recorded daily for 10 days. Dead grasshoppers were removed and immediately deposited in high-humidity chambers (sterile Petri dishes with filter paper dampened with sterile distilled water). Mycosis was confirmed by microscopical examination of the dead grasshoppers.

## Results and Discussion

**Figure 2.**
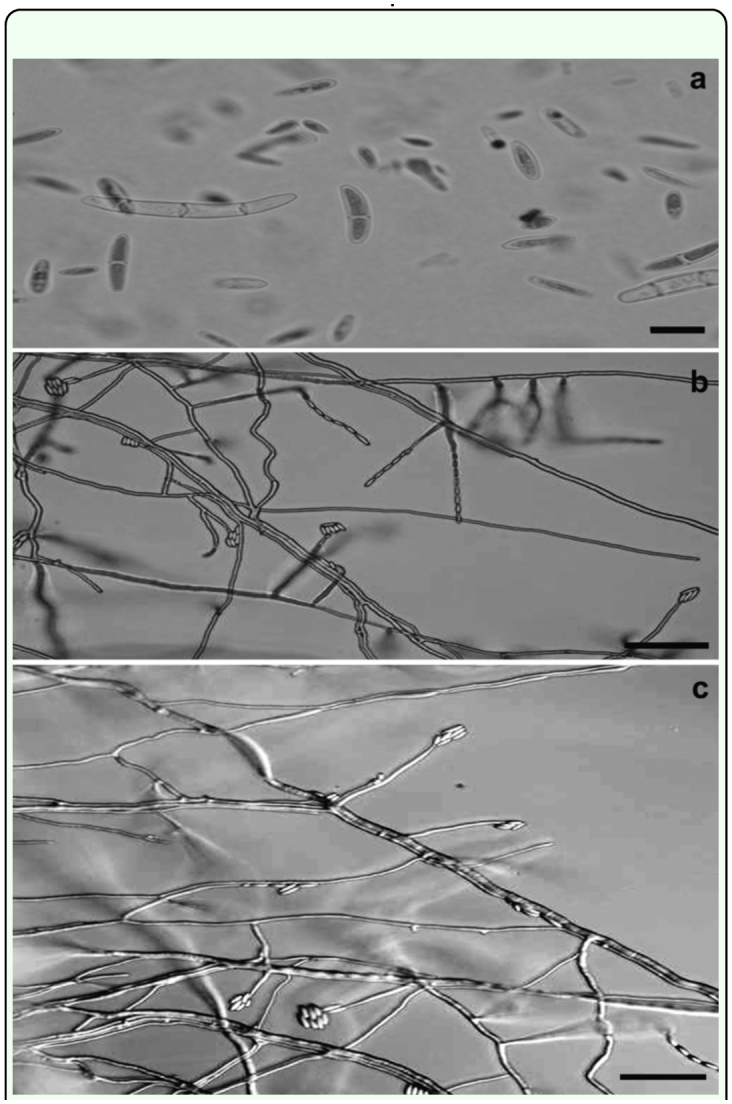
(a) Macro- and microconidia of *Fusarium verticillioides* (b and c) Phialides and microconidia in chain and grouped in heads. Scale bar: (a) 10 µm; (b and c) 30 µm. High quality figures are available online.

The isolated fungus was morphologically identified as *F. verticillioides* on carnation leaf-piece agar and on PDA according to Nelson et al. (1983) and Leslie and Summerell (2006). Their characteristics on the former agar were: macroconidia, formed in paleorange sporodochia, slightly falcate or else straight with thin walls, and the basal cell foot-shaped, 3–5 septate, 25–55 × 2–4 µm (Figure 2a); microconidia, formed in chains on monophialides, oval to club-shaped with a flattened base, usually 0 septate, 6–10 × 1.5– 2.5 µm (Figure, 2b, c). Chlamydospores were not found. Characters on PDA were: white aerial mycelium with rapid growth, violet pigmentation with age; lower surface with violet to dark-violet pigmentation.

The isolate determined to be *F. verticillioides* on the basis of morphological characteristics produced a PCR-amplified fragment of 800-bp identical to that observed by Patino et al. (2004).

**Figure 3.**
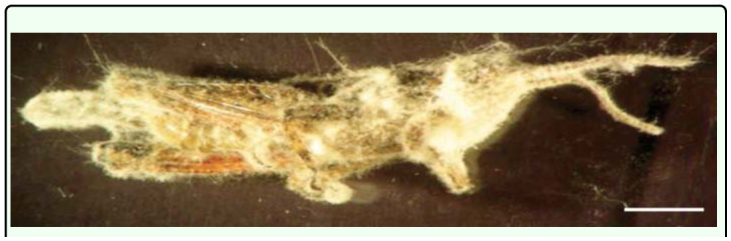
*Fusarium verticillioides* on third-instar nymphs of *Ronderosia bergi* 48 h after death. Scale bar: 14 mm. High quality figures are available online.

The average viability of *F. verticillioides* conidia was 95%. The mortality caused by *F. verticillioides* on *R. bergi* reached 58 ± 6.53% by 10 days after inoculation. Phialides and conidia were observed growing out of the dead hosts (Figure 3). No mortality occurred among the controls.

Majumdar et al. (2008) obtained higher mortality rates in laboratory tests reaching 80% mortality with the pupae of *Tetanops myopaeformis* 10 days after applying the same concentration (2.8 × 10^6^) of *Fusarium solani* conidia. An even greater mortality was observed by Golpalakrishnan and Narayan (1989), who reported 100% in the guavashield scale, *Pulvinaria* (formerly known as *Chloropulvinaria*) *psidii* Maskel, (Hemiptera: Coccidae) at 5 days after treatment with 4.8 × 10 conidia/ml of *Fusarium oxysporum*. Moreover, a 100% mortality was obtained within three days in a field-cage test with *F. oxysporum* against *Nilaparvata lugens* (Kuruvilla and Jacob 1979a, 1980).

The means by which the infection of *T. collaris* and *R. bergi* by *F. verticillioides* might have been effected is not clear, but Kilpatrick (1961) stated that the entry of the fungus into the insect could occur via the oral route, oviposition tubes or wounds.

Relatively low temperatures and high moisture levels have been shown to be suitable for the development of *Fusarium* epizootics in other insects (Villacaros and Robin 1989; Venugopal et al. 1989; Pandit and Tarannum 2002; Majumbar et al. 2008). Such conditions do not normally occur in the area sampled by us where the temperatures are much higher (42–46° C) and the rainfall lower (600 mm annually).

At present we do not know if *T. collaris* would be affected by the fungus in the field. However, such possibility should not be ruled out. Although low humidity and elevated temperature are usually unfavorable for most fungi, this particular isolate was recovered living under the conditions affecting the host. Perhaps the fungus is one of the factors preventing further spread of the host into wetter, cooler regions.

In conclusion, the present study provides the first report of *F. verticillioides* as an entomopathogenic fungus in grasshoppers and extends the knowledge of the pathogenic capacity of this *Fusarium* species to that orthopteran. Future research efforts should investigate the possible existence of mycotoxin production against humans and the potential effects of the *F. verticillioides* isolate LPS 1057 on nontarget fauna. Moreover, future pathogenic studies may clarify whether or not this isolate is capable of infecting plants and ascertain by molecular phylogenetic analyses if this isolate has an identical, similar, or somewhat altered germ-line-genetic linage to that of the *F. verticillioides* plant-pathogens.
